# Traditional Chinese herbal medicine-potential therapeutic application for the treatment of COVID-19

**DOI:** 10.1186/s13020-020-00419-6

**Published:** 2021-02-22

**Authors:** Jillian L. Capodice, Barbara M. Chubak

**Affiliations:** Department of Urology, Icahn School of Medicine, Mount Sinai Health System, New York, NY USA

**Keywords:** TCM, Chinese medicine, Herbal medicine, COVID-19, COVID

## Abstract

Traditional Chinese Medicine (TCM) is a complete medical system that has evolved over millennia to include practices and procedures such as acupuncture, herbal medicine, manual therapies, nutrition, and mind–body therapies such as *qi gong*. In modern-day China and other Asian countries, TCM is a medical subspecialty utilized alongside western biomedicine. During the current Coronavirus Disease 19 (COVID-19) pandemic, TCM and TCM herbal medicine is being used and a number of single herbs and combination formulas have significant bioactivity and therapeutic potential. The purpose of this paper is to highlight the use of TCM in the treatment of COVID-19. This commentary provides the reader with a concise background on COVID-19 and summarizes TCM concepts including identification, pattern diagnosis, and treatment principles commonly used for the treatment of viral influenza-like diseases. It also highlights some of the challenges and potential for using TCM in an integrated medical setting.

## Background

### COVID-19: Epidemiology and Clinical characteristics

A novel coronavirus disease identified in 2019 was named by the World Health Organization following a series of reported pneumonia cases in Wuhan, the capital of Hubei province in China, in December 2019 [1, 2]. Coronavirus disease 19 (COVID-19) was subsequently classified and renamed, Severe Acute Respiratory Syndrome Coronavirus 2 (SARS-CoV-2), by the International Committee on Taxonomy of Viruses. COVID-19 was formally characterized as a pandemic on 11 March 2020 [1, 2]. In the literature and clinic, the terms COVID, COVID-19, COVID-positive, and/or SARS-CoV-2 are frequently used interchangeably.

Coronaviruses are helical ribonucleic acid (RNA)-based viruses that have a large genome. They are capable of infecting humans and have a range of other mammalian hosts besides humans, including bats, and birds [2]. Coronaviruses are named for their spikey surface proteins that resemble a crown (Latin *corona*); these are one of several structural proteins [3, 4]. The SARS-CoV-2 virus is thought to enter the cell via angiotensin converting enzyme 2, a transmembrane protein in type-2 pneumocytes that are found in the alveoli, nasal goblet cells, and intestinal epithelial cells [2–5]. It is thought that the virus preferentially infects the type-2 pneumocytes in the lungs, but may concurrently infect intestinal epithelial cells [5].

Transmission of COVID occurs through respiratory droplet cells and likely via aerosolization and asymptomatic spread [6, 7]. Viral replication of COVID in humans triggers innate and adaptive immune responses promoting an influx of activated neutrophils and inflammatory macrophages, monocytes, and antibodies [8]. COVID-positive patients present with a wide variety of common influenza (flu)-like symptoms, and patients may present with some but not all of the following symptoms: fever, dry cough, sore throat, myalgia, fatigue, shortness of breath, nasal congestion, headache, chills, diarrhea, nausea, vomiting. While the majority of patients infected with COVID-19 suffer from mild flu-like symptoms that commonly resolve within a few days up to a couple weeks, all patients—but particularly those with chronic cardiovascular conditions, type-2 diabetes, chronic respiratory diseases, cancer, and people above 60 years of age—are at risk of a more severe disease course and even death [9]. Progressive and moderate to severe COVID-19 symptoms include worsening pneumonia that may lead to acute respiratory distress syndrome, respiratory failure, acute cardiac injury, and heart failure. Additional complications include sepsis, acute kidney injury, acidosis, and risk of secondary infections, all of which can result in a serious disease course and/or death [9–11].

### Therapeutic options for the treatment of COVID-19

At present there are no curative treatments for COVID-19. Treatment is symptomatic and supportive, and may include oxygen therapy, mechanical ventilation, hemodynamic support, medical treatment with antimicrobial, antiviral, or other pharmaceutical drugs, as well as the interventional and medical management of other complications in the patient with severe COVID [9, 10, 12]. In many countries, including China, France, Italy, and the United States, patients may also receive off-label, compassionate-use therapies, or clinical trial therapies including antivirals, aminoquinoline antimalarial, convalescent plasma, steroid, anti-interleukin-6 inhibitors, and other pharmacologic drug therapies [13–17]. Recently a number of vaccines have been approved for emergency use in the US and countries such as the United Kingdom and France. These are based on interim data from clinical trials of newly developed vaccines [18, 19]. In China and other Asian countries that utilize TCM, there have also been studies of TCM therapies and herbal medicine for the treatment of COVID-19 [20–24]. When applying TCM herbal medicine, familiarity with TCM theory is required. This paper provides the reader with a concise background on TCM herbal medicine theory and practice and discusses its potential in the treatment armamentarium for COVID-19.

### Traditional Chinese medicine principles

The clinical application of traditional Chinese herbal medicine requires a thorough understanding of TCM theory. TCM herbal medicines are comprised of any number of primarily botanical ingredients that are combined together in a formula and administered to patients as treatment for symptoms and diagnoses according to TCM. For the reader unfamiliar with TCM this means the patient has both a biomedical diagnosis and a TCM pattern diagnosis. Traditional Chinese herbal medicine has evolved over millennia, during which the Chinese materia medica have been continually added to and modified by doctors of traditional medicine. In the modern-day People’s Republic of China (PRC), TCM is commonly used both alone and in combination with western biomedical pharmaceutical medicines and treatments. Similar branches of traditional Asian medicine are used in South Korea, Japan, and other Asian countries [25].

The most common route of administration of TCM herbal formulas is oral, and oral delivery forms include teas, decoctions, granules, tablets, and capsules. Frequently used formulas are also available as premade patent formulas (usually tablets or capsules) [26]. In China and other Asian countries, TCM is a medical subspecialty commonly utilized in the healthcare system in hospital, academic, and ambulatory settings and during the COVID-19 pandemic, TCM herbal medicine (along with procedures to treat symptoms such as acupuncture) is being used in combination with biomedical pharmacologc and other medical approaches as supportive care treatment for COVID patients [21, 22].

### Pattern diagnosis of viral influenza-like disorders according to TCM

The treatment of viral flu-like disorders has been recognized for a long time in TCM. The foundational text that established core clinical theories on the treatment of flu-like illnesses is, The Discussion of Cold-Induced Disorders (*Shang han za bing lun* commonly called *Shang Han Lun*), written by Zhang Zhong-jing at the end of the Han Dynasty (206 BCE–220 CE). This text was utilized for many years until the emergence of the Warm-Febrile School or *Wen Bing* School in the eighteenth century. The most important text from the *Wen Bing* School is, Systematic Differentiation of Warm Diseases (*Wen bing tiao bian*), and modern TCM theory and herbal formulas derived and adapted from this text are commonly used to treat acute viral flu-like symptoms and disorders [26].

Foundations of TCM diagnosis includes clinical presentation identification, pattern diagnosis, and application of the appropriate therapy based on treatment principles. Prophylaxis of diseases and treatment adaptation/individualization are core tenets of TCM practice whether herbal medicines or procedures are used alone or in combination with biomedical treatments. For the treatment of patients with COVID-19, a few national Chinese treatment guidelines were released containing TCM therapeutics. For recommended TCM herbal medicines in the guidelines, there are slight variations in the formulas and treatment principles but variations are not uncommon in the context of TCM or general clinical practice.

The main clinical presentation stages of COVID-19 include: prevention stage (prevention and asymptomatic patients), early-stage (mild flu-like symptoms), later-stage (pneumonia symptoms), and recovery (convalescence) [21–23]. Once a patient’s clinical presentation is classified, a pattern diagnosis is then made. For early stage presentations some common patterns include sore throat, fever, and cough (called pattern 1 for the purposes of this paper). Pattern 2 includes pattern 1 symptoms plus headache and myalgia, and pattern 3 includes aforementioned symptoms with nausea, vomiting, or diarrhea. With each pattern diagnosis, an appropriate TCM herbal medicine and other treatments are prescribed. The treatment principle corresponding to early-stage pattern 1 symptoms is to ‘release the exterior’. The treatment principle of ‘releasing the exterior and clearing heat’ is suggested for pattern 2, and the principles of ‘dispelling dampness and reducing toxic heat’ are used for pattern 3. In patients who present or progress to later-stage, pneumonia-like symptoms, treatment principles include ‘eliminating damp-heat’, ‘reducing toxic stagnation’, and/or ‘rescuing the exterior’. TCM treatment principles and herbal formulas used for patients recovering from COVID-19 are designed to ‘supplement and heal the lungs’ or ‘restore fluids and support lung and spleen *qi* (energy)’ [21–24]. Table 1 outlines TCM Principles for the treatment of the flu-like symptoms and patterns commonly seen in symptomatic patients with COVID-19.

**Table 1:** TCM principles and pattern diagnosis for the treatment of viral influenza-like disorders

Clinical stage	Common clinical symptoms	Pattern diagnosis	Treatment principle
Prevention	Healthy or Asymptomatic	Not applicable	Support normal *qi*, supplement the organs
Early	Mild symptoms including sore throat, cough, fever, headache, myalgia, nausea and vomiting, diarrhea, shortness of breath	Wind cold	Release the exterior
		Wind heat	Release the exterior and clear heat
		Intermittent cold and heat	Dispel dampness
Late	Any/all of the above with worsening pneumonia symptoms and worsening of laboratory and/or radiographic findings	Damp heat obstructing the lung	Eliminate damp-heat
		Internal blockage of epidemic toxin	Reduce toxic stagnation
		Closed interior and abandoned exterior	Rescue the exterior
Recovery	Sequelae of symptoms	Qi and yin deficiency	Supplement and heal the lungs
		Deficiency of lung and spleen	Restore fluids and support lung and spleen

### Utility of TCM in COVID, research challenges, and advancing TCM

The COVID-19 treatment guidelines and TCM principles used by practitioners of TCM in China and throughout the world underscore the utility and promise of TCM as medical treatment and underscores the continued challenges of research and TCM clinical practice in an integrated medical setting. There have been a few case series’ describing the TCM therapies used in the treatment of patients with COVID-19 such as those as described by Ren et al. and Wan et al. [27, 28]. Ren et al. reported detailed data characterizing 102 COVID cases in patients who presented with mild symptoms who were treated with medicines including TCM. The authors reported that “clinical symptom disappearance time was shortened by 2 days, the recovery time of body temperature was shortened by 1.7 days, the average length of stay in hospital was shortened by 2.2 days” in patients with early stage symptoms. The authors concluded that early intervention with TCM herbal medicine may delay disease progression and improve cure rates, offering various potential mechanisms of action to explain the formulas’ helpfulness such as, “Treatment practice of COVID-19 showed that early intervention of TCM is important way to improve cure rate, shorten the course of disease, delay disease progression and reduce mortality rate. Furthermore, the reason why TCM works is not only to inhibit the virus, but might block the infection, regulate the immune response, cut off the inflammatory storm, and promote the repair of the body. Moreover, the prevention and control measures of COVID-19 fully reflect the ideology of ‘preventive treatment of disease’” [27, 29]. Wan et al. recently published a case series describing the management of Chinese COVID-19 patients from Hubei. This analysis of 135 COVID-positive hospitalized patients in Chongqing (a region northeast to Wuhan in Hubei) demonstrated that 135 [100%] patients received antiviral therapy, 59 [43.7%] received antibacterial therapy, 36 [26.7%] received corticosteroids, and 124 [91.8%] received TCM [28].

These series show the clinical course and treatment of patients with COVID using TCM and also highlights some of the single herbal ingredients that are used in the formulas. It also brings to light the challenges that are faced when studying TCM herbal medicine. From a biomedical pharmacologic reductionist approach, the study of single ingredients or individual molecular compounds continues to be the gold standard, and allows us to look at the mechanism of action of a single agent. For example the molecular analysis of single herbs such as Radix scutellariae (common name-skullcap or *Huang qin*) and Radix astragali (common name- astragalus or *Huang qi)* demonstrate that these botanicals have broad antiviral activity in-vitro [30, 31]. Compounds found in Radix scutellariae, such as the flavonoid baicalin and other phytochemicals, have antiviral activity and have been shown to reduce autophagosome formation via reduction of cellular lipids [32]. A recent study demonstrated inhibition of H1N1 influenza viral activity by flavonoids extracted from Radix scutellariae [33]. Radix astragali has been shown to contain bioactive molecules including alkaloids, flavonoids, saponins, and triterpene glycosides and extracts of Radix Astragali have been shown to exert pharmacologic activity on the cardiovascular, hepatic, immune, and respiratory systems [34–36]. A recent comprehensive review by Zhang et al. describes its traditional use, chemical constituents, and some of its pharmacological effects. For example, Radix Astragali has been shown to enhance humoral and cellular immunity in animal models, with potent antiviral activity in-vitro and in-vivo [34–36].

Research that evaluates complete TCM herbal formulas rather than single herbs is also of particular importance. Li et al. recently published results of an in-vitro examination of the antiviral activity of *Lian hua qing wen* (LH), a TCM patent medicine comprised of 13 herbs, which is a recommended TCM COVID treatment according to the PRC Guideline [22, 23]. In the in-vitro analysis the results demonstrated that treatment of Vero E6 cells with LH resulted in an inhibition of SARS-CoV-2 replication and a corresponding reduction in the pro-inflammatory cytokines, tumor necrosis factor-alpha, interleukin-6, monocyte chemoattractant protein 1 (CCL-2/MCP-1) and CXCL-10/IP-10 at the mRNA level [37]. Another in-vitro study screened and identified TCM botanicals with potential to act against SARS-CoV-2 by inhibiting proteins essential to viral entry and replication, such as papain-like protease and spike protein. The authors screened and isolated several molecules from a wide variety of TCM herbs and demonstrated that these may have potent antiviral and immune modulating properties and further research is needed to confirm whether these isolated molecules behave similarly when combined together [38].

Faced with these unknowns and because of the current coronavirus pandemic, we are compelled to ask: how can we start to perform controlled clinical trials of agents such as TCM herbal medicines without reducing these formulas to a single molecule? Can the integrity of TCM medicine, which emphasizes the idiosyncrasy of its individual patients, be maintained in the face of rigorous randomized clinical trial design, which reduces its subjects to homogenized comparison groups? A recent viewpoint written by Dr. Andre Kalil comments on the importance of utilizing adaptive clinical trial designs during a pandemic, that are “able to rapidly accept or reject multiple experimental therapies throughout the trial, while being adequately powered for meaningful clinical outcomes” [13]. Could a TCM herbal formula be included as a potential therapy to be tested?

## Conclusions

COVID-19 is an ongoing pandemic with no curative treatment. Treatment of hospitalized patients with symptomatic COVID relies on supportive care, including oxygenation, mechanical ventilation, hemodynamic support, and management of comorbidities and complications. Examples of proposed therapies for the treatment of COVID include antiviral, aminoquinoline antimalarial, antimicrobial, interleukin-6, and plasma-based treatments that are currently being tested for their safety and effectiveness in the treatment of advanced pneumonia, acute respiratory distress, and other effects of COVID infection. New vaccines have been developed and approved for emergency use in many countries. Traditional Chinese Medicine herbal formulas are comprised of numerous ingredients that are most commonly taken orally. They are formulated and applied based on TCM principles including clinical stage presentation identification, pattern diagnosis selection, and treatment application based on TCM treatment principles. TCM herbal medicine has been used in China to treat COVID-19 patients with various symptoms across a range of disease risk and severity. Positive outcomes of TCM treatment have been reported, but similarly to western biomedicines, continual monitoring of adverse events and safety are essential for all herbal and drug agents. Individual herbs and their phytoactive components have shown antiviral and immune modulation properties in research laboratory settings, and serious consideration should be given to further, rigorous scientific study of promising TCM herbal medicines outside of China, as potential treatments for this disease.

## Data Availability

Not applicable.

## Abbreviations

TCM: Traditional Chinese Medicine
COVID-19: Coronavirus Disease-19
SARS-CoV-2: Severe Acute Respiratory Syndrome Coronavirus 2
COVID: Coronavirus Disease
PRC: People’s Republic of China
RNA: Ribonucleic acid

## References

[1] Zhou P, Yang X, Wang X (2020). A pneumonia outbreak associated with a new coronavirus of probable bat origin. Nature.

[2] Zhu N, Zhang D, Wang W (2020). A novel coronavirus from patients with pneumonia in China, 2019. N Engl J Med.

[3] Walls A, Park YJ, Tortorici MA (2020). Structure, function, and antigenicity of the SARS-CoV-2 spike glycoprotein. Cell.

[4] Andersen KG, Rambaut A, Lipkin WI (2020). The proximal origin of SARS-CoV-2. Nat Med.

[5] Xiao F, Tang M, Zheng X (2020). Evidence for gastrointestinal infection of SARS-CoV-2. Gastroenterology.

[6] Cai J, Sun W, Huang J (2020). Indirect virus transmission in cluster of COVID-19 cases, Wenzhou, China, 2020. Emerg Infect Dis..

[7] Guo YR, Cao QD, Hong ZS, Tan YY, Chen SD, Jin HJ, Tan KS, Wang DY, Yan Y (2020). The origin, transmission and clinical therapies on coronavirus disease 2019 (COVID-19) outbreak - an update on the status. Mil Med Res..

[8] Huang C, Wang Y, Li X (2020). Clinical features of patients infected with 2019 novel coronavirus in Wuhan, China. Lancet.

[9] Guan WJ, Ni ZY, Hu Y, Liang WH, Ou CQ, He JX (2020). Clinical characteristics of coronavirus disease in China. N Engl J Med..

[10] Severe Outcomes Among Patients with Coronavirus Disease 2019 (COVID-19)—United States, February 12–March 16, 2020. MMWR Morb Mortal Wkly Rep. ePub. DOI: 10.15585/mmwr.mm6912e2 10.15585/mmwr.mm6912e2 PMC7725513 32214079

[11] Clinical course and risk factors for mortality of adult inpatients with COVID-19 in Wuhan, China: a retrospective cohort study. Lancet 2020. 10.1016/S0140-6736(20)30566-3 PMC7270627 32171076

[12] Matthay MA, Aldrich JM, Gotts JE (2020). Treatment for severe acute respiratory distress syndrome from COVID-19. Lancet Respir Med..

[13] Kalil AC (2020). Treating COVID-19—Off-label drug use, compassionate use, and randomized clinical trials during pandemics. JAMA.

[14] Gautret P, Lagier JC, Parola P (2020). Hydroxychloroquine and azithromycin as a treatment of COVID-19: results of an open-label non-randomized clinical trial. Int J Antimicrob Agents.

[15] Gao J, Tian Z, Yang X (2020). Breakthrough: Chloroquine phosphate has shown apparent efficacy in treatment of COVID-19 associated pneumonia in clinical studies. Biosci Trends.

[16] Yuen KS, Ye ZW, Fung SY, Chan CP, Jin DY (2020). SARS-CoV-2 and COVID-19: The most important research questions. Cell Biosci..

[17] Rajendran K, Krishnasamy N, Rangarajan J, Rathinam J, Natarajan M, Ramachandran A (2020). Convalescent plasma transfusion for the treatment of COVID-19: systematic review. J Med Virol..

[18] Polack FP, Thomas SJ, Kitchin N, Absalon J, Gurtman A, Lockhart S, Perez JL, Pérez Marc G, Moreira ED, Zerbini C, Bailey R, Swanson KA, Roychoudhury S, Koury K, Li P, Kalina WV, Cooper D, Frenck RW, Hammitt LL, Türeci Ö, Nell H, Schaefer A, Ünal S, Tresnan DB, Mather S, Dormitzer PR, Şahin U, Jansen KU, Gruber WC, C4591001 Clinical Trial Group (2020). Safety and Efficacyof the BNT162b2 mRNA Covid-19 Vaccine. N Engl J Med..

[19] Jackson LA, Anderson EJ, Rouphael NG, Roberts PC, Makhene M, Coler RN, McCullough MP, Chappell JD, Denison MR, Stevens LJ, Pruijssers AJ, McDermott A, Flach B, Doria-Rose NA, Corbett KS, Morabito KM, O'Dell S, Schmidt SD, Swanson PA, Padilla M, Mascola JR, Neuzil KM, Bennett H, Sun W, Peters E, Makowski M, Albert J, Cross K, Buchanan W, Pikaart-Tautges R, Ledgerwood JE, Graham BS, Beigel JH, mRNA-1273 Study Group (2020). An mRNA Vaccine against SARS-CoV-2 - Preliminary Report. N Engl J Med..

[20] Cunningham AC, Goh HP, Koh D (2020). Treatment of COVID-19: old tricks for new challenges. Crit Care.

[21] Handbook of COVID-19 Prevention and Treatment. The First Affiliated Hospital, Zhejiang University School of Medcine. Editor-in-chief: Liang T. https://www.elotus.org/promo-files/COVID-19_resources/Handbook_of_COVID_19_Prevention_en_Mobile.pdf

[22] Guidance for Corona Virus Disease 2019, Prevention, Control, Diagnosis and Management. Edited by National Health Commission of the PRC, Compiled and Translated by Chinese Preventive Medicine Association. https://www.elotus.org/promo-files/COVID-19_resources/Guidance%20for%20Corona%20Virus%20Disease%202019%20(English).pdf

[23] Chen J. Coronavirus (COVID-19) Treatment with TCM in China. Accessed 26 May 2020 https://www.elotus.org/promo-files/COVID-19_resources/How%20Coronavirus%20(Covid-19)%20is%20treated%20with%20TCM%20in%20China%20by%20John%20K%20Chen%20v3.pdf.

[24] Chan KW, Wong VT, Tang SCW (2020). COVID-19: An Update on the epidemiological, clinical, preventive and therapeutic evidence and guidelines of integrative chinese-western medicine for the management of 2019 novel coronavirus disease. Am J Chin Med.

[25] World Health Organization. WHO global report on traditional and complementary medicine 2019. World Health Organization. https://apps.who.int/iris/handle/10665/312342. License: CC BY-NC-SA 3.0 IGO. 2019.

[26] Bensky D. Chinese herbal medicine: formulas & strategies. seattle, wash: Eastland Press, 1990.

[27] Ren JL, Zhang AH, Wang XJ (2020). Traditional Chinese medicine for COVID-19 treatment. Pharmacol Res.

[28] Wan S, Xiang Y, Fang W (2020). Clinical features and treatment of COVID-19 patients in Northeast Chongqing. J Med Virol.

[29] Publicity Department of the People’s Republic of China. Press conference of the joint prevention and control mechanism of state council on Feb 17, 2020. http://www.nhc.gov.cn/xcs/fkdt/202002/f12a62d10c2a48c6895cedf2faea6e1f.shtml. Accessed Feb 23, 2020.

[30] Wu Y, Wang X, Xue J (2017). Plant phenolics extraction from Flos Chrysanthemi: response surface methodology based optimization and the correlation between extracts and free radical scavenging activity. J Food Sci.

[31] Han X, Zhang DK, Guo YM (2016). Screening and evaluation of commonly-used anti-influenza Chinese herbal medicines based on anti-neuraminidase activity. Chin J Nat Med.

[32] Wang MJ, Yang CH, Jin Y (2020). Baicalin inhibits Coxsackievirus B3 replication by reducing cellular lipid synthesis. Am J Chin Med.

[33] Zhi HJ, Zhu HY, Zhang YY (2019). In vivo effect of quantified flavonoids-enriched extract of *Scutellaria baicalensis* root on acute lung injury induced by influenza A virus. Phytomedicine.

[34] Zhang CH, Yang X, Wei JR (2019). Ethnopharmacology, phytochemistry, pharmacology, toxicology and clinical applications of radix astragali. Chin J Integr Med.

[35] Liang Y, Zhang Q, Zhang L (2019). Astragalus membranaceus treatment protects Raw2647 cells from influenza virus by regulating G1 phase and the TLR3-mediated signaling pathway. Evid Based Complement Alternat Med..

[36] Kallon S, Li X, Ji J (2013). Astragalus polysaccharide enhances immunity and inhibits H9N2 avian influenza virus in vitro and in vivo. J Anim Sci Biotechnol.

[37] Runfeng L, Yunlong H, Jicheng H (2020). Lianhuaqingwen exerts anti-viral and anti-inflammatory activity against novel coronavirus (SARS-CoV-2). Pharmacol Res.

[38] Zhang DH, Wu KL, Zhang X (2020). In silico screening of Chinese herbal medicines with the potential to directly inhibit 2019 novel coronavirus. J Integr Med.

